# Associations Between Myeloid-Derived Suppressor Cells, TIM-3+ T Cells, and Clinical Factors During the Post-transplant Neutropenia Period in Patients With Multiple Myeloma

**DOI:** 10.7759/cureus.86641

**Published:** 2025-06-24

**Authors:** Egor Batorov, Tamara Tyrinova, Tatyana Aristova, Vera Denisova, Dariya Batorova, Svetlana Sizikova, Galina Ushakova, Aleksandr Ostanin, Elena Chernykh

**Affiliations:** 1 Laboratory of Cellular Immunotherapy, Research Institute of Fundamental and Clinical Immunology, Novosibirsk, RUS; 2 Department of Immunology, Vladimir Zelman Institute for Medicine and Psychology, Novosibirsk National Research State University, Novosibirsk, RUS; 3 Laboratory of Cellular and Molecular Mechanisms of Immunopathology, Research Institute of Fundamental and Clinical Immunology, Novosibirsk, RUS; 4 Department of Hematology and Bone Marrow Transplantation, Research Institute of Fundamental and Clinical Immunology, Novosibirsk, RUS

**Keywords:** carbapenems, granulocyte colony-stimulating factor, hematopoietic stem cell transplantation, multiple myeloma, myeloid-derived suppressor cells, tim-3

## Abstract

Introduction: The objective of our study was to assess relationships between circulating myeloid-derived suppressor cell (MDSC) populations and T cell subsets, up-regulating PD-1 and TIM-3, as well as their intended association with several post-transplant clinical factors.

Methods: Forty-five patients with multiple myeloma (MM) were enrolled in the study. Circulating Lin^-^HLA-DR^-^CD33^+^CD66b^+ ^polymorphonuclear (PMN) MDSCs, CD14^+^HLA-DR^low/- ^monocytic (M) MDSCs, PD-1^+^/TIM-3^+^ CD4^+^ and CD8^+^ T cells were assessed with flow cytometry at the engraftment and following six post-transplant months. The frequencies of patients with common post-transplant complications, antibacterial treatment, and granulocyte colony-stimulating factor (G-CSF) administration during the neutropenia period were investigated.

Results: TIM-3^+^ CD4^+^ and CD8^+^ T cell counts were negatively associated with M-MDSCs at the engraftment. Both PD-1^+^/TIM-3^+ ^T cells and MDSCs at the engraftment did not differ between the patients with or without febrile episodes, oral mucositis, and enteropathy during the neutropenia period. Relative counts of PMN-MDSCs were significantly lower in MM patients who received carbapenems compared to those treated with cefepime. Short-term G-CSF administration was associated with an increase in M-MDSCs.

Conclusion: Following autologous hematopoietic stem cell transplantation (HSCT), cellular interactions and the possible impact of therapeutic interventions on the immune recovery have not been fully investigated and may be of interest as predictive biomarkers and targets for novel therapies.

## Introduction

Multiple myeloma (MM) is a malignant disease of unknown origin characterized by the uncontrollable proliferation of clonal B cell precursors that further differentiate into monoclonal immunoglobulin-producing plasma cells within the bone marrow. The abnormal plasma cells undermine physiological bone remodeling, leading to bone lesions, impairment of blood cell production, pathological fractures, and hypercalcemia, while monoclonal immunoglobulins cause kidney injury. This relatively frequent hematological malignancy is still considered incurable. Despite impressive advancements in novel anti-myeloma therapeutic approaches (from proteasome inhibitors to anti-BCMA CAR T cells), high-dose melphalan (HDM) with autologous hematopoietic stem cell transplantation (HSCT) remains a cornerstone in the treatment for eligible patients, aiming to achieve sustained remission and improve overall survival rates. Apart from the anti-tumor effect, the myeloablative conditioning with HDM irreversibly impairs hematopoiesis. Subsequent critical pancytopenia, chemotherapy-induced inflammation, frequent post-transplant infectious complications, and re-infusion of the autologous graft containing both mature immune cells and hematopoietic precursors launch an avalanche-like production and release of a wide range of cytokines. As a result, following a one-time graft re-infusion, virtually all immune cell populations begin to reconstitute synchronously but with different dynamics. Previously, it was shown that the timely recovery of distinct lymphoid and myeloid cell populations was associated with post-transplant survival rates following both autologous and allogeneic HSCT. The favorable associations were established for cell compartments involved in anti-tumor immune response: lymphocytes [1,2], NK cells [3,4], and classical CD14^+^CD16^-^ monocytes [5]. However, the functional state of cytotoxic cells matters: exhausted/senescent CD8^+^ T cells were found to be increased in patients with MM prior to post-transplant relapse [6]. Several research groups have reported the transient increase in T cells up-regulating immune checkpoint receptors PD-1, TIM-3, TIGIT, and others following HSCT [7-9]. Immune checkpoints play pivotal roles in maintaining immune homeostasis but also contribute to a state known as "T cell exhaustion," characterized by attenuation of anti-tumor activity. On the other hand, these activated and/or dysfunctional T cells are considered promising targets for therapeutic intervention at post-transplant [6,7].

Both anti- and pro-tumor immune cells of myeloid lineage reconstitute much faster compared to lymphocytes [10]. Recently, we have shown a rapid increase in myeloid-derived suppressor cell (MDSC) subsets after HDM with autologous HSCT [11]. MDSCs play a crucial role in tumor growth, promoting angiogenesis, facilitating malignant cell survival, and creating an immunosuppressive microenvironment through the secretion of various cytokines and induced enzymes (interleukin-10 (IL-10), transforming growth factor-β (TGF-β), arginase-1, indoleamine 2,3-dioxygenase, and inducible nitric oxide synthase) [12]. Of note, higher frequencies of MDSCs at post-transplant might be linked both to decreased and increased progression-free survival [11,13].

The impact of MDSCs on the counts and functional properties of lymphocytes under the conditions of immune recovery is of particular interest, but still not fully studied. Understanding and targeting these interactions hold promise for enhancing the immune response post-autologous HSCT.

The objective of our present study was to assess relationships between circulating MDSC populations and T cell subsets, up-regulating inhibitory receptors PD-1 and TIM-3, as well as their intended association with several post-transplant clinical factors in patients with MM following HDM with autologous HSCT.

## Materials and methods

Forty-five patients with MM who had been treated with the HDM conditioning with autologous HSCT at the Department of Hematology and Bone Marrow Transplantation (BMT) of the Research Institute of Fundamental and Clinical Immunology (Novosibirsk, Russia) from May 2018 to June 2022 were enrolled in the study. All patients gave informed consent in accordance with the Declaration of Helsinki of 1975; the local ethics committee approved the study protocol. The patients were staged according to the Durie-Salmon system (1975) [14]. Responses were defined according to the International Myeloma Working Group Criteria [15]. Baseline characteristics of patients are described in Table 1.

**Table 1:** Baseline characteristics of patients. Age and hematopoietic progenitor cell dose are presented as medians (min-max). The remaining data are presented as individual values. Percentages are calculated from the total number of patients (n=45).

Characteristic		Value
Age at the conditioning, years		54 (37-72)
Sex, female/male		27/18 (60%/40%)
Types	IgG kappa/IgG lambda	24/6 (53%/13%)
	IgA kappa/IgA lambda	4/1 (9%/2%)
	Light chain	4 (9%)
	Non-secretory	1 (2%)
	No data	5 (11%)
Durie-Salmon stage	II	14 (31%)
	III	31 (69%)
Disease status at analysis	Complete remission, very good partial response	23 (51%)
	Partial response	22 (49%)
Induction therapy regimens	1 (bortezomib-based regimens)	28 (62%)
	2 (bortezomib-based regimens + DCEP/EDAP/lenalidomide-containing regimens)	15 (33%)
	≥3 (bortezomib-based regimens + DCEP/EDAP + lenalidomide-containing regimens)	2 (4%)
CD45^+^CD34^+^ hematopoietic progenitor cell dose, × 10^6^/kg		4.35 (2.2-13.8)

For hematopoietic progenitor cell (HPC) mobilization, the patients received either high-dose cyclophosphamide (4 g/m²) or, rarely, a regular induction course followed by granulocyte colony-stimulating factor (G-CSF) injections (5 μg/kg/day for four to six days). Apheresis sessions (n=1-2) were performed with ASTEC 204 (Fresenius Medical Care, Germany) or Spectra LRS 07 (COBE Spectra Apheresis System, USA) cell separators until ≥2.0 × 106 CD34^+^CD45^+^ HPCs/kg were collected. Prior to HPC re-infusion, patients received pre-transplant conditioning with HDM (140-200 mg/m²).

Peripheral blood samples

A total of 70 peripheral blood (PB) samples were obtained from 45 patients with MM during routine diagnostic procedures at the engraftment (n=45) and following six months (n=25). PB samples (8 mL) were collected in vacuum tubes and processed within two hours of collection. Mononuclear cells (MNCs) were isolated by Ficoll density gradient (ρ=1.077 g/mL) centrifugation, washed twice with phosphate buffer solution, and underwent flow cytometric analysis within 24 hours.

Flow cytometric analysis

**Figure 1:**
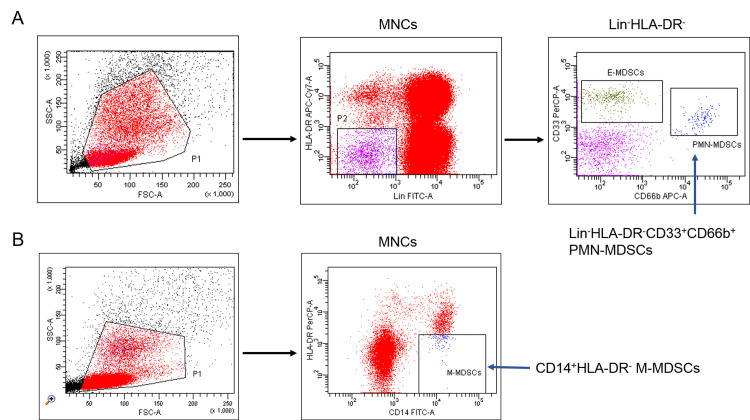
Flow cytometric characteristics of the studied myeloid-derived suppressor cell populations. Gating strategies for Lin^–^HLA-DR^–^CD33^+^CD66b^+^ polymorphonuclear myeloid-derived suppressor cells (PMN-MDSCs) (A) and CD14^+^HLA-DR^low/–^ monocytic myeloid-derived suppressor cells (M-MDSCs) (B) are shown. Data of a representative multiple myeloma patient are presented. For analysis, 245634 and 193871 events in the MNC gate (P1) were acquired for PMN-MDSC (A) and M-MDSC (B) assessment, respectively.

**Figure 2:**
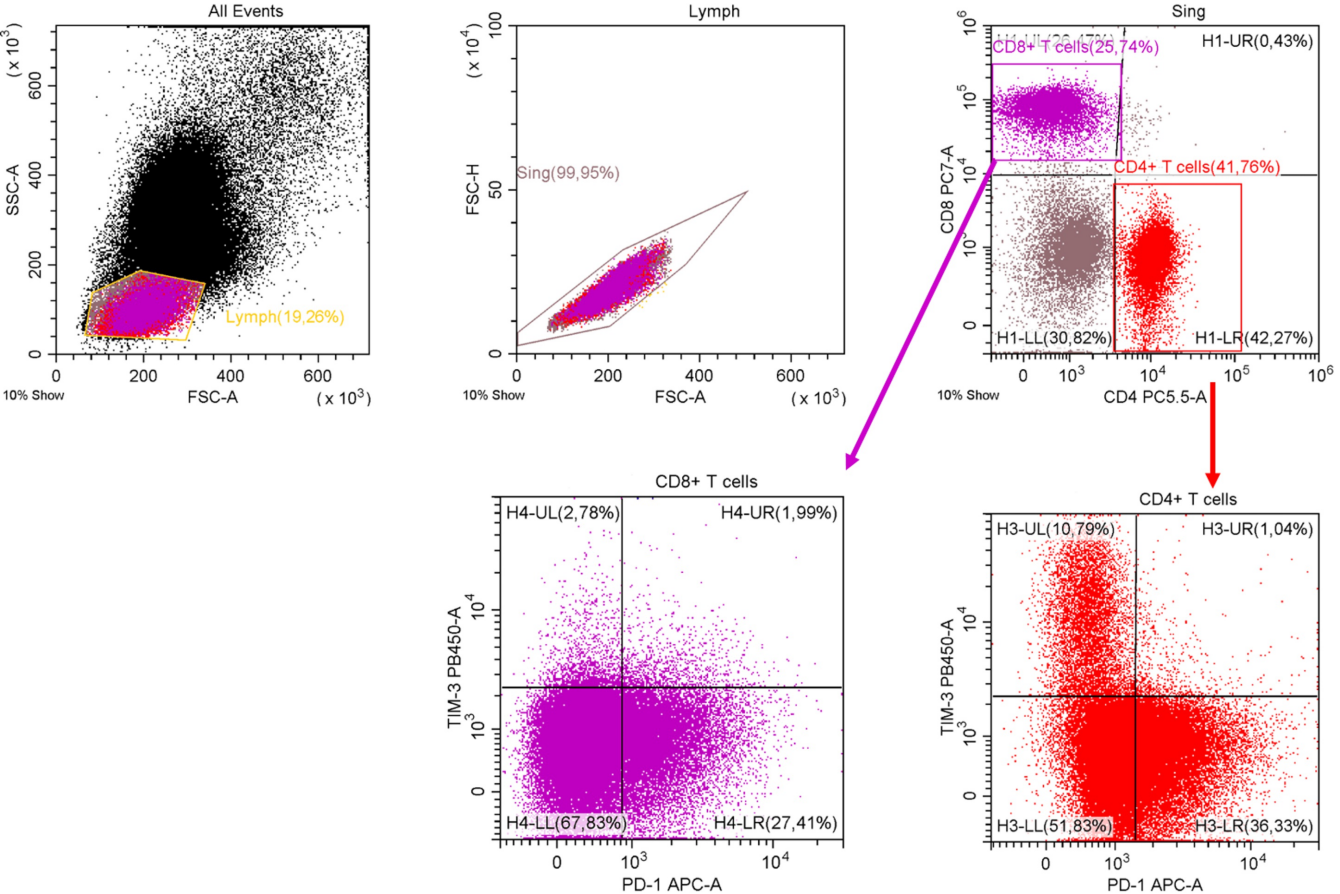
Flow cytometric characteristics of PD-1+ and TIM-3+ T cell subsets. Lymphocyte gate was selected. Cell doublets were excluded from the analysis based on characteristics of forward scatter area (FSC-A) and height (FSC-H). Relative counts of CD4^+^, CD8^+^ T cells expressing PD-1 and TIM-3 were studied in appropriate regions. Data of a representative multiple myeloma patient are presented. For analysis, 99776 and 59320 events were acquired in CD4^+^ and CD8^+^ T cell gates, respectively.

The following populations were assessed with multiparametric flow cytometry: Lin^-^HLA-DR^-^CD33^+^CD66b^+^ polymorphonuclear MDSCs (PMN-MDSC), CD14^+^HLA-DR^low/-^ monocytic MDSCs (M-MDSC), CD4^+^PD-1^+^, CD4^+^TIM-3^+^, CD8^+^PD-1^+^, CD8^+^TIM-3^+^, CD4^+^PD-1^+^TIM-3^+^, CD8^+^PD-1^+^TIM-3^+^ T cells. Monoclonal antibodies used are listed below: anti-Lineage Cocktail 1 (CD3, CD14, CD16, CD19, CD20, CD56; FITC, BD Pharmingen™, San Jose, CA, USA), anti-CD14 (FITC; BD Pharmingen™), anti-HLA-DR (PerCP, APC-Cy7; BD Pharmingen™), anti-CD33 (PerCP-Cy5.5; BD Pharmingen™), anti-CD66b (APC; BD Pharmingen™), anti-CD8 (PE-Cy7; BD Pharmingen™), anti-CD4 (PerCP; San Diego, CA, Biolegend, USA), anti-TIM-3 (BV421; BD Pharmingen™), anti-PD-1 (APC; BD Pharmingen™). As controls were used unstained live cells and “fluorescence-minus-one” samples. Samples were analyzed on a FACSCanto II flow cytometer with FACSDiva software (BD Biosciences, USA) and DxFLEX (Beckman Coulter, USA). Gating strategies are presented in Figures 1, 2.

Statistical analysis

Statistical analysis was performed using Statistica 6 (StatSoft, Inc., Tulsa, OK, USA) and GraphPad Prism 5.0 (GraphPad Software, Inc., La Jolla, CA, USA) software. Data in the text were presented as median and interquartile ranges unless otherwise specified. The Mann-Whitney U test was used to calculate differences between groups. Spearman’s rank correlation and linear regression models were performed to evaluate associations for continuous variables. P-values presented were two-sided. P < 0.05 was considered statistically significant.

## Results

**Figure 3:**
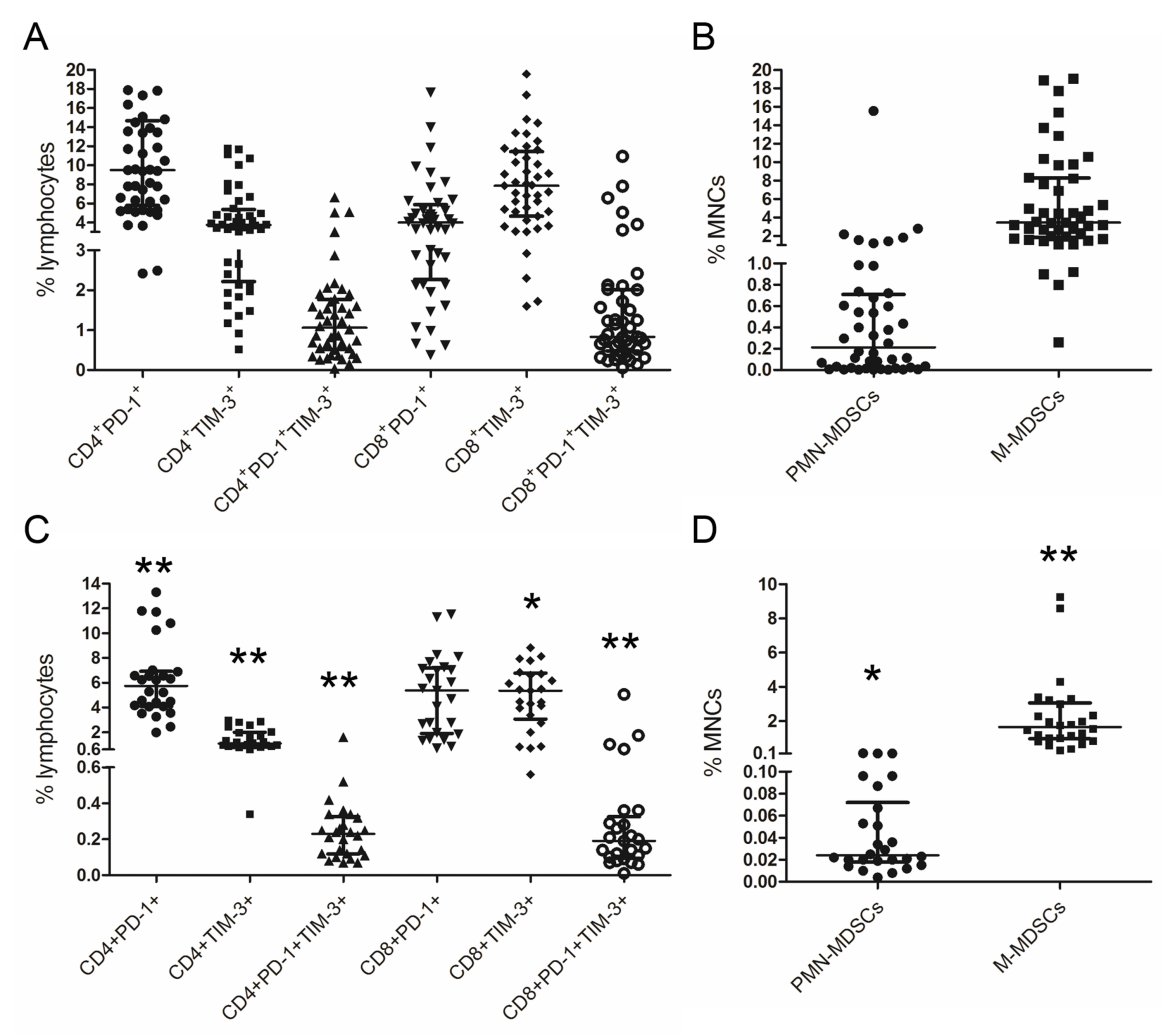
Relative counts of PD-1+ and TIM-3+ T cells and myeloid-derived suppressor cells in patients with multiple myeloma at post-transplant. Circulating PD-1^+^ and TIM-3^+^ T cells (A, С), Lin^–^HLA-DR^–^CD33^+^CD66b^+^ polymorphonuclear MDSCs (PMN-MDSCs) and CD14^+^HLA-DR^low/–^ monocytic MDSCs (M-MDSCs) (B, D) in patients with MM at the engraftment day (n=45) (A, B) and following six to 12 months after high-dose chemotherapy (n=25) (C, D) are presented. Data are expressed as individual values; lines represent median ± interquartile range values. P-values are assessed with the Mann–Whitney U-test. Significant differences between cell populations at the engraftment and following six to 12 post-transplant months are indicated as asterisks (*P_U_<0.005, **P_U_<0.0005).

Previously, we and several other research groups had shown the increase in MDSC populations [11,16] and PD-1 and TIM-3 expressing T cell subsets [7-9] following high-dose chemotherapy with HSCT compared to their pre-transplant counterparts. The frequencies of T cell subsets and MDSCs that were assessed in the present study are shown in Figure 3. 

Besides, previously we failed to find associations between circulating MDSCs and PD-1^+^/TIM-3^+^ T cells, except for the weak correlation between CD14^+^HLA-DR^low/-^ M-MDSCs and CD8^+^PD-1^+^ T cells in 60 patients with MM in remission [17]. At the engraftment, the frequencies of circulating TIM-3-expressing CD4^+^ and CD8^+^ T cells were negatively correlated with M-MDSCs (Table 2). There were no associations between the evaluated T cell subsets and monocyte percentages (Table 2), as soon as M-MDSCs were not correlated with absolute lymphocyte counts: r_S_=0.14, p=0.35; N=45.

**Table 2:** Correlation analysis between PD-1+ and TIM-3+ T cells and myeloid-derived suppressor cells in patients with multiple myeloma at early post-transplant. Data from 45 patients with MM were analyzed. Correlation analysis was performed between relative counts of PD-1^+^/TIM-3^+^ T cells and MDSCs presented as the percentages of lymphocytes and MNCs, respectively. r_S_ – Spearman’s rank correlation; MM: multiple myeloma; MDSCs: myeloid-derived suppressor cells; PMN: polymorphonuclear; M: monocytic; MNCs: mononuclear cells

Lymphocyte subset	PMN-MDSCs, %	Monocytes, %	M-MDSCs, %
CD4^+^PD-1^+^, %	r_S_=-0.29; p=0.057	r_S_=-0.050; p=0.75	r_S_=-0.049; p=0.75
CD4^+^TIM-3^+^, %	r_S_=0.12; p=0.44	r_S_=-0.053; p=0.74	r_S_=-0.40; p=0.0070
CD4^+^PD-1^+^TIM-3^+^, %	r_S_=-0.018; p=0.91	r_S_=-0.068; p=0.66	r_S_=-0.50; p=0.00048
CD8^+^PD-1^+^, %	r_S_=0.19; p=0.23	r_S_=-0.10; p=0.52	r_S_=-0.25; p=0.099
CD8^+^TIM-3^+^, %	r_S_=0.010; p=0.95	r_S_=0.0023; p=0.98	r_S_=-0.37; p=0.015
CD8^+^PD-1^+^TIM-3^+^, %	r_S_=0.17; p=0.27	r_S_=0.0019; p=0.91	r_S_=-0.37; p=0.016

The relatively slight but significant associations of M-MDSC and TIM-3^+^ T cell relative counts at early post-transplant were confirmed in the linear regression models (Table 3). 

**Table 3:** Associations between M-MDSCs and TIM-3+ T cell subset counts in patients with multiple myeloma (n=45) at the engraftment in linear regression models. Results of linear regression analyses to predict frequencies of TIM-3^+^ T cell subsets at the engraftment with relative M-MDSC count as an independent variable. A forward stepwise ridge linear regression model was used. MDSCs: myeloid-derived suppressor cells; M: monocytic

Lymphocyte subset (dependent variable)	Regression results with M-MDSCs, %, as an independent factor			
	R^2^, coefficient of determination	β-coefficient of regression	Fisher criterion	P
CD4^+^TIM-3^+^, %	0.12	–0.34	5.7	0.022
CD4^+^PD-1^+^TIM-3^+^, %	0.10	–0.32	4.8	0.035
CD8^+^TIM-3^+^, %	0.11	–0.33	4.9	0.032
CD8^+^PD-1^+^TIM-3^+^, %	0.10	–0.32	4.8	0.035

Following six to 12 months post-transplant, relative counts of almost all studied cell populations (except CD8^+^PD-1^+^ T cells) were significantly declined compared to their counterparts at the engraftment (Figure 3C, 3D). At that time point, the negative correlations between TIM-3^+^ T cells and M-MDSCs were lost; the only association was between CD8^+^PD-1^+^ T cells and PMN-MDSCs: r_S_=0.45, p=0.014; N=25.

Thus, M-MDSC frequency was negatively associated with TIM-3-expressing T cell subsets at early post-transplant but not later.

Relative content of polymorphonuclear myeloid-derived suppressor cells at the engraftment lower in patients with multiple myeloma treated with carbapenems during the cytopenia period

Next, we evaluated possible clinical factors that could affect the studied T cell and MDSC counts. During the period of post-transplant cytopenia following the conditioning and autograft re-infusion, most patients experienced toxic and infectious complications and received antimicrobial therapy (Table 4).

**Table 4:** Complications occurred during the period of post-transplant cytopenia and their management. G-CSF: granulocyte colony-stimulating factor

Characteristic		No of patients
Microbiologically or clinically documented infections, fever of unknown origin, no/yes		5/40
Oral mucositis, grades	No	7
	1	18
	2	13
	3	6
	4	1
Enteropathy, grades	No	20
	1	4
	2	15
	3	6
Antibiotics	None	4
	The 1^st^ line (cefepime)	26
	The 2^nd^ line (carbapenem)	15
Metronidazole, no/yes		30/15
G-CSF administration, days	0	30
	1	12
	≥2	3

Relative counts of PD-1-, TIM-3-expressing T cells and MDSC populations at the engraftment did not differ between the patients with or without febrile episodes, oral mucositis, and enteropathy during the neutropenia period (data not shown).

**Figure 4:**
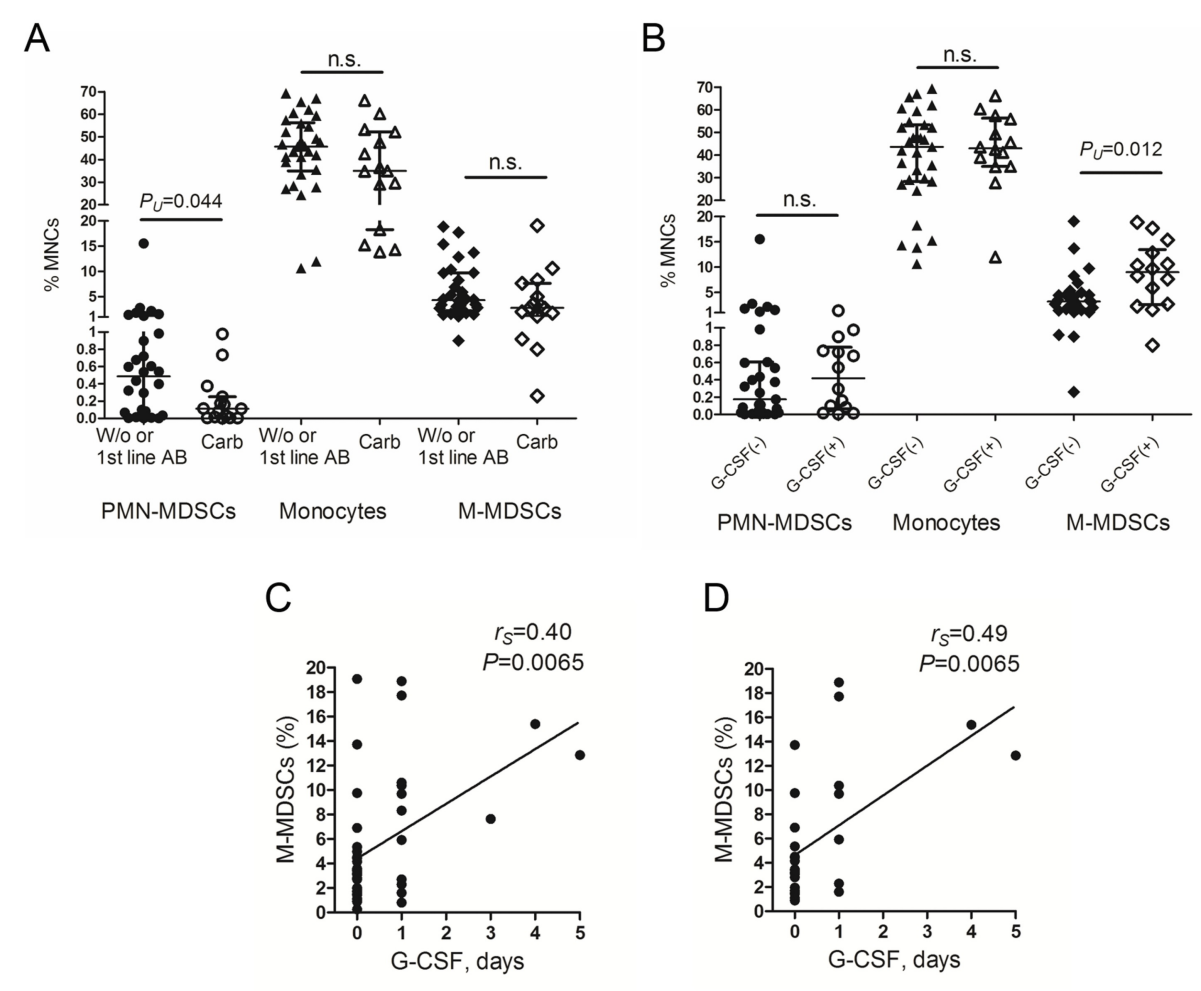
Monocytes and myeloid-derived suppressor cells at the engraftment depending on the therapy administered during the cytopenia period. Graphs show relative counts of circulating Lin^–^HLA-DR^–^CD33^+^CD66b^+^ polymorphonuclear myeloid-derived suppressor cells (PMN-MDSCs), monocytes, and CD14^+^HLA-DR^low/–^ monocytic MDSCs (M-MDSCs) at the time of cytopenia recovery. The patients were separately divided depending on the following treatment during the cytopenia period: (A) without antibacterial therapy and treated with the first line antibiotics (“W/o or 1st line AB”, black symbols; n=30) or received carbapenems (“Carb”, empty symbols; n=15), and (B) with or without G-CSF administration (“G-CSF(+)”, empty symbols; n=15, and “G-CSF(-)”, black symbols; n=30, respectively). Correlations between M-MDSC frequencies at the engraftment and the duration of G-CSF administration in the entire group of patients (n=45) (C) and following the exclusion of the individuals treated with carbapenems (n=30) (D). Data are expressed as individual values; lines represent median ± interquartile range values (A, B). P values are assessed with the Mann–Whitney U-test (A, B). Correlations were evaluated with the Spearman rank correlation test (C, D). G-CSF: granulocyte colony-stimulating factor

At the time of the study, the standard first-line antibiotic for empiric treatment of infectious complications in our clinic was cefepime, while the second line was represented by carbapenems. Thirteen patients received meropenem, and two were treated with imipenem. Frequencies of PMN-MDSCs were significantly lower in the patients received carbapenems (N=15) comparing with the rest ones (i.e., treated with the first line antibiotic only or without any antibacterial therapy, N=30): 0.11% (0.006-0.25%) vs 0.54% (0.029-1.10%); P_U_=0.044 (Figure 4A).

Absolute PMN-MDSC count was also decreased in the carbapenem group as a trend: 0.6 /μL (0.1-3.0 /μL) vs 3.4 /μL (0.7-10.5 /μL); P_U_=0.083. Despite the similar direction of changes, M-MDSC counts in the same groups did not significantly differ (Figure 4A).

There were no differences in the studied cell populations between the patients who received or did not receive metronidazole (data not shown).

Granulocyte colony-stimulating factor administration during the cytopenia period associated with an increase in monocytic myeloid-derived suppressor cells at the engraftment

G-CSF medications can be administered following hematopoietic stem and progenitor cell transplantation (HSPCT) to speed up neutrophil recovery. In our study, 15 patients were treated with G-CSF during the cytopenia period (300 μg/day); most of them (N=12) received a single injection (median day +15 (+14-+16); only non-pegylated drugs were used). Despite the short course of treatment, relative M-MDSC count was significantly increased in the patients following G-CSF administration (N=15) compared with the non-treated individuals (N=30) (Figure 4B). Absolute M-MDSC count was also higher in G-CSF-treated patients (as a trend): 79.2 /μL (19.0-255.4 /μL) vs 20.3 /μL (14.3-54.0 /μL); P_U_=0.052.

The frequency of M-MDSCs and the duration of G-CSF therapy were significantly correlated (Figure 4C). The correlation was also confirmed for absolute M-MDSC content: r_S_=0.34, p=0.029; N=45. The number of G-CSF administration days was associated with M-MDSC count in a linear regression model: R^2^=0.22, β=0.46; P=0.0015, F-criterion=11.5; P < 0.0015, n=45.

Since carbapenems appear to affect the frequency of M-MDSCs, we excluded the data of patients receiving these antibiotics during the cytopenia period. The adjustment led to further improvement of the regression model: R^2^=0.30, β=0.55; P=0.0019, F-criterion=11.8; P < 0.0019, n=30. Figure 4D also shows correlation analysis of the adjusted data. Among the studied lymphocyte populations, relative CD8^+^TIM-3^+^ T cell count only was negatively correlated as a trend with G-CSF duration: r_S_=-0.27, p=0.075; N=45.

## Discussion

Rapid immune recovery following both autologous and allogeneic HSCT seems to be associated with improved survival rates [10], and therefore continues to be under scrutiny. Apart from quantitative prevalence in anti-tumor immune cell populations, their functional properties appear to be of great importance. T cells upregulate inhibitory receptors PD-1, TIM-3, and LAG-3 under conditions of homeostatic proliferation following HSCT [8,9]. Expansion and cytotoxic activity of lymphocytes is under the control of various suppressive cells that are traditionally considered pro-tumor: regulatory T cells (Tregs), MDSCs, innate lymphoid cells, tumor-associated M2 macrophages, tolerogenic dendritic cells, N2-neutrophils, mast cells, stromal cells [18]. Among the listed cell populations, at least Tregs and MDSCs have previously been shown to recover quickly after HSCT [11,16,19,20]. Cellular interactions under the conditions of immune recovery at post-transplant are of particular interest as possible predictive biomarkers and potential targets for novel therapies. The aim of our present study was to investigate relationships between circulating MDSCs and T cells, expressing inhibitory receptors PD-1 and TIM-3 in patients with MM following HDM with autologous HSCT. We also assess associations with distinct post-transplant clinical factors and the study cell populations.

We and other research groups previously have shown the increase in both PD-1 and TIM-3 expressing T cell subsets [7-9] and MDSC populations [11,16] following high-dose chemotherapy with HSCT compared to their pre-transplant values. Under lymphopenic conditions, the increment in actively proliferating and functional T cells up-regulating PD-1 and TIM-3 can be stimulated with common cytokine receptor γ-chain family cytokines - IL-2, IL-7, IL-15, IL-21, - involved in homeostatic proliferation of mature lymphocytes [21,22]. Up-regulation of checkpoint inhibitory receptors during cytokine-induced peripheral expansion may be required to hinder the proliferation of inflammatory or autoaggressive T cells. At late post-transplant, however, the increase in PD-1^+^ and TIM-3^+^ T cells had been associated with early relapse [6,8].

Nonetheless, we have shown that M-MDSC frequency was negatively associated with TIM-3^+^ and PD-1^+^TIM-3^+^ T cell subsets at the engraftment. We failed to find such correlations between circulating MDSCs and PD-1^+^/TIM-3^+^ T cells in patients with MM both before HDM [17] and following six months post-transplant. According to previous studies, the changes in MDSC and checkpoint receptor-expressing T cell content in various malignancies are predominantly unidirectional [23]. MDSCs are able to induce the expression of PD-1, TIM-3, LAG-3, and TIGIT. Besides, MDSCs realize their suppressive potential through checkpoint ligands PD-L1, galectin-9, and CD155 (for PD-1, TIM-3, and TIGIT, respectively) [24].

The detected negative associations between M-MDSCs and CD4^+^TIM-3^+^ and CD8^+^TIM-3^+^ T cells (including PD-1^+^TIM-3^+^ subsets) might be a consequence of MDSC suppressive function. Following allogeneic HSCT, elevated levels of MDSCs have been associated with graft-versus-host disease (GVHD) alleviation (reviewed in [25]). Concerning autologous HSCT, expanded MDSCs may attenuate the potential reduction in the diversity of the T cell receptor (TCR) repertoire at post-transplant by preventing the proliferation of re-infused mature T cells. According to the data of Pinton et al, activated T cells facilitated an increment and suppressive potential of MDSCs via the IL-10 production. MDSCs, in turn, regulated T cell functional properties through the B7-H1-PD-1 and MHC II-LAG-3 interactions and arginase-1 and indoleamine 2,3-dioxygenase production [26]. On the contrary, J. Chen et al. found a negative correlation between activated T cells (measured by co-expression of tumor necrosis factor-α (TNFα) and interferon-γ) and MDSC counts and functional features (viability and arginase-1 production) in human colorectal cancer specimens following anti-PD-1 treatment. In a series of in vitro assays, T cells after activation were able to decline MDSC content and suppressive potential via interferon-α/β-mediated TNF-related apoptosis-inducing ligand (TRAIL)-TRAIL receptor 2 interaction, additionally stimulated with TNFα [27]. The cross-talk between MDSCs and reconstituted lymphocyte populations is a complex process and needs further thorough investigations.

Next, we evaluated the possible impact of several clinical factors on the content of the studied T cell and MDSC counts. During the period of post-transplant cytopenia following HDM and autograft re-infusion, most patients experienced febrile episodes, oral mucositis or enteropathy, and received appropriate medications. We found that PMN-MDSC content at early post-transplant was significantly lower in the patients who received carbapenems compared with those without antibacterial therapy and treated with cefepime during the cytopenia period. SE Lee et al. showed lower ratios of early-stage MDSCs and M-MDSCs to T cells in patients who had received carbapenems following allogeneic HSCT. Simultaneously, the use of carbapenems has been associated with both the loss of intestinal microbiota diversity and the increased occurrence of intestinal GVHD. The authors presumed that altered intestinal microbiota might be less effective at expanding MDSC populations; the latter ones subsequently lost control of GVHD [28].

In our study, PMN-MDSC decline was associated with carbapenem treatment, while M-MDSCs did not statistically differ (2.80% (1.13-7.64%, N=15) vs. 4.16% (2.30-9.70%, N=30); P_U_= 0.27), presumably, due to relatively low number of observations or stimulating effect of G-CSF. However, the identified similarities in the changes of MDSC content following carbapenem administration in patients with MM may be of interest. Previously, Khan et al. showed that the decline in gut bacterial diversity and skew to distinct taxa in patients who underwent autologous HSCT were reminiscent of those with allogeneic HSCT. Increased fecal intestinal diversity in the cytopenia period was associated with a lower risk of death or progression [29]. Following autologous HSCT, changes in gut microbiome and interplay between regulatory cell populations (MDSCs, Tregs, etc) and convenient T cells rarely take such dramatic forms as GVHD and therefore are still under-researched.

Next, we found the increment in M-MDSCs following short-term G-CSF administration in patients with MM at the engraftment. The frequency of M-MDSCs was associated with the duration of G-CSF injections, especially following adjustment to carbapenem usage. The elevation of MDSC populations in response to G-CSF stimulation in vivo is anticipated and has been described previously during HSC mobilization [11,30]. At the same time, the statistically insignificant increase in PMN-MDSCs following G-CSF was a bit unexpected. We speculate that PMN-MDSCs at post-transplant have already exhibited rapid proliferation due to intense release of myeloid-stimulating cytokines, and it's possible that a brief series of additional G-CSF injections (actually, a single one in most cases) had no impact.

G-CSF is extensively utilized in the management of hematological disorders, particularly for the mobilization of HSCs and the reduction of neutropenia duration. The use of G-CSF after autologous and allogeneic HSCT is generally considered safe [31,32]. Thus, there are no apparent adverse immunosuppressive effects from the transient G-CSF-mediated MDSC elevation. Nonetheless, such consequences of colony-stimulating factors treatment should be taken into account; e.g., McCartney et al, also showing the increase in MDSCs following G-CSF administration, appealed to avoid its uncontrolled use at post-transplant [30].

The limitation of our investigation is that we did not evaluate the post-transplant recovery of Tregs. It was not the scope of the study; nonetheless, it would be interesting to estimate counts of the most relevant suppressor cell populations simultaneously. In our study, we did not examine the composition and diversity of intestinal microbiota, and may just speculate regarding any crosstalk between broad-spectrum antibiotics, gut microbiome, and MDSCs following autologous HSCT. More research is necessary on this subject, which is typically underappreciated and understudied. Another limitation is the absence of data on bone marrow T cells and MDSCs at early post-transplant, but this source is not available closely after HSCT.

## Conclusions

TIM-3^+^ CD4^+^ and CD8^+^ T cell counts were negatively associated with M-MDSCs at the engraftment. Putatively, M-MDSCs could hinder the expansion of T cell clones that gain an advantage at early post-transplant.

Both PD-1^+^/TIM-3^+^ T cells and MDSCs at the engraftment did not differ between the patients with or without febrile episodes, oral mucositis, and enteropathy during the neutropenia period. Relative counts of PMN-MDSCs were significantly lower in patients who received carbapenems compared to those without antibacterial therapy or treated with cefepime. Presumably, altered intestinal microbiota are less effective at expanding MDSCs. Short-term G-CSF administration was associated with an increase in M-MDSCs. The frequency of M-MDSCs was associated with the duration of G-CSF injections.
